# Do Cross-National and Ethnic Group Bullying Comparisons Represent Reality? Testing Instruments for Structural Equivalence and Structural Isomorphism

**DOI:** 10.3389/fpsyg.2019.01621

**Published:** 2019-08-23

**Authors:** Muthanna Samara, Mairéad Foody, Kristin Göbel, Mohamed Altawil, Herbert Scheithauer

**Affiliations:** ^1^Department of Psychology, Kingston University London, Kingston upon Thames, United Kingdom; ^2^Anti-Bullying Research Centre, Dublin City University, Dublin, Ireland; ^3^Department of Education and Psychology, Freie Universität Berlin, Berlin, Germany; ^4^Department of Psychology, University of Hertfordshire, Hafield, United Kingdom; ^5^Palestine Trauma Centre (PTC-UK), Hertfordshire, United Kingdom

**Keywords:** bullying, victimization, cross-national comparison(s), ethnic differences, structural equivalence, structural isomorphism

## Abstract

Bullying in schools is a widespread phenomenon, witnessed worldwide, with negative consequences for victims and perpetrators. Although it is an international issue, there are several issues with cross-national and cross-cultural/ethnic research that can make comparisons between countries and cultures/ethnic groups difficult including language, cultural perception, and/or methodological issues. As statistical techniques rapidly develop, there may be more scope to be statistically creative in how we assess the utility of one tool across different groups such as cultures, nations, etc. At the very least, an attempt to do this should be paramount in studies investigating different groups (e.g., from different countries) at one time. This study investigated bullying and victimization rates in a large cross-ethnic and -country comparison between adolescents from four countries and five different ethnic groups including: Israel (Jewish Israelis and Arab Palestinian Israelis), Palestine (the Gaza Strip), Germany, and Greece. A total of 3,186 school children aged 12–15 years completed self-report questionnaires of peer bullying/victimization. A stepwise data analytic approach was used to test comparability of the psychometric properties: (1) Structural equivalence contributes to the valid use of the instrument in cultural contexts other than the one for which the instrument has been developed. Structural equivalence is a necessary condition for the justification of indirect or direct comparisons between cultural groups. (2) Additionally, structural isomorphism is necessary to demonstrate that the same internal structure of the instrument applies to the cultural and individual levels. Findings support the internal structural equivalence of the questionnaire with the exception of the Palestinian sample from the Gaza Strip. Subsequently, exploratory factor analysis on the cultural level structure revealed a one-factor structure with congruence measure below 0.85. Thus, no evidence was found for internal structural isomorphism suggesting that no direct comparisons of cultural samples was justified. These results are discussed in detail and the implications for the international research community and cross-national/-ethnic comparison studies in bullying are addressed.

## Introduction

Bullying is a specific form of aggressive behavior that includes repeated and negative behavior patterns (e.g., intentional injury) by one or several individuals toward another. In addition, the definition of bullying includes a real or perceived imbalance in power where the victim cannot defend him/herself (Olweus, 1994). International research suggests that bullying is a widespread phenomenon with similar characteristics across various countries and cultures globally. For example, gender differences are evident with regard to direct or physical bullying and victimization (boys are more involved than girls) and victimization usually decreases when pupils grow older (e.g., Scheithauer et al., 2006; Smith et al., 2019). Being actively involved in bullying represents a major threat to healthy development and is associated with maladjustment later in life (e.g., Wolke et al., 2012; Zwierzynska et al., 2013; Slava et al., 2018). In particular, students who report bullying behavior as well as victimization (bully-victims), have a higher risk of developing emotional and behavioral problems (Wolke and Samara, 2004; Winsper et al., 2012; Kennedy, 2018; for a summary see Hess and Scheithauer, 2015).

Apart from similarities, cross-national and ethnic cultural research on bullying has produced numerous studies comparing prevalence rates and impact worldwide (e.g., Borntrager et al., 2009; Craig et al., 2009; Ortega et al., 2012; Chester et al., 2015; Smith et al., 2016a; Athanasiou et al., 2018). One study by Fleming and Jacobsen (2009) compared bullying rates in 19 countries worldwide using data from the Global School-based Student Health Survey. Results showed Zambia as the country with the highest percentage of victims (60.9%) and Tajikistan as the lowest (7.8%). Although the instruments used were the same for each country, the authors noted that interpretations were unique to each culture group and that social stigma could account for discrepancies across the countries. Another cross-national study by Mark et al. (2013) compared bullying rates in Lithuania, Luxemburg, and Estonia and showed that Lithuanian boys accounted for the biggest percentage of bullies, while girls in Luxemburg accounted for the smallest.

Indeed, several longitudinal studies have emerged which make comparisons of bullying involvement over time and across several countries such as the EU Kids Online study (e.g., Livingstone et al., 2015) or the Health Behavior in School Aged Children study (HBSC; e.g., Zaborskis et al., 2018; for a summary Smith and López-Castro, 2017). These studies are worthwhile in terms of drawing comparisons of bullying prevalence across many countries, yet they do not come without their difficulties. For example, individual countries often report varying rates for victimization across these studies and the studies themselves have shown limited comparability (Smith et al., 2016b; Smith and López-Castro, 2017).

There are several issues with cross-national and cross-ethnic cultural research that can make comparisons between countries and cultures or ethnic groups difficult. The first major issue of research is to ensure the psycholinguistic equivalence of the term “bullying.” Notably, in some countries (e.g., Italy) no adequate translation of the English word “bullying” exists. In addition, there is no Arabic term equivalent to bullying (Samara et al., unpublished) and as such there is much debate about the most appropriate word to use and differences between one or more related concepts on bullying (Scheithauer et al., 2016). Moreover, even when the language is the same, there is the problem of varying terms to explain bullying-related behavior such as peer harassment or aggression. This is an issue both within a country as well as between countries (Smorti et al., 2003). On the same note, interpretation of what constitutes other types of bullying (e.g., cyberbullying) and the importance of definitional elements (e.g., anonymity) has been shown to vary across countries (Menesini et al., 2012). Other important factors when conducting this type of research refer to methodological issues that can also differ across studies and limit comparisons that can be drawn. These include research instruments used, the time frame questions refer to (e.g., the last 6 months vs. 12 months vs. the past term), and even if a definition is provided or not (Foody et al., 2017). Not only are there methodological differences in how questionnaires are delivered and what they enquire about (e.g., time frame), there are more general cultural differences that the instrument may not be sensitive to (e.g., what it means to be a bully and the social implications of such) that could be related to social desirability and cultural norms.

Several other non-methodological factors can also determine country differences, such as socioeconomic inequality (Chaux and Castellanos, 2015) or cultural values (e.g., individualism–collectivism; Smith and Robinson, 2019). For example, a cross cultural study amongst 75 countries revealed less overall victimization in individualist societies but greater proportion of relational victimization and a higher ratio of bullies to victims in collectivist societies (Smith and Robinson, 2019).

### Comparability Across Ethnic Groups: Psychometric Properties of Tools Used in Cross-National/Cultural Studies on Bullying

For the most part, researchers use a mix of strategies in trying to ensure their tools transfer across cultures such as translation and back translation of questions, factor analysis of items, and inclusion and exclusion of explanations in various languages. For example, several new scales have been developed to investigate cyberbullying across several countries. For the most part, strict statistical methods are used such as exploratory and confirmatory factor analysis. As statistical techniques rapidly develop, there may be more scope to be statistically creative in how we assess the utility of one tool across cultures and nations. At the very least, an attempt to do this should be paramount in studies investigating many countries at once.

When administering a psychometric instrument in a questionnaire-based survey in different cultural or ethnic groups with the aim to compare the groups on a particular scale, we need first to test the respective instrument for its comparability across different cultural or ethnic groups as these comparisons could be misleading. There are three main reasons why this is the case. Firstly, this could be due to the cultural specificity of the instrument. Cultural systems can determine the meaning and characteristics of a specific psychological construct and process (Miller, 1997), which can differ between different ethnic and national groups (e.g., individualist societies vs. collectivist societies can generate different meaning for the same bullying instrument and thus different quantitative results).

Secondly, there may be distorting effects relating to methodological biases affecting specific items (e.g., translation biases and errors) or possibly the whole instrument (e.g., due to culturally different perceptions in relation to response styles), lack of familiarity with the testing procedure, underrepresentation of the construct domain by the content of the test (e.g., other forms of victimization are missing) and so on. These methodological biases could violate the conditions for equivalent metric and/or structure across cultures and thus, quantitative cross-cultural comparisons could produce misrepresentative results.

Thirdly, there may be a lack of generalizability of individual-level constructs to the national/cultural level. It could be that a specific construct (e.g., victimization) is used to describe individuals within a specific culture or ethnic group but does not necessarily characterize the national group as a whole. Thus, for example, when a bullying/victimization questionnaire is used with a specific cultural group and generates total scores of bullying and victimization, these scores describe and represent the characteristics of the individuals in the cultural group. When we then compare between ethnic/national groups based on these total scores or constructs, these scores become representative of these ethnic/national groups and we then assume cross-cultural differences. However, referring and attributing these individual-level characteristics to ethnic and national groups as a whole is misleading as the meaning of that specific bullying and/or victimization construct can alter from the individual level to the cultural one (Matsumoto and Van de Vijver, 2010).

As a result, the relation between specific scale items and the underlying dimensions may change across different (cultural) groups. It is therefore necessary to investigate the equivalence of the internal structure in each new ethnic or cultural group where the instrument is applied. A stepwise data analytic approach is suggested by Fischer and Fontaine (2010) and Fontaine and Fischer (2010) to test the comparability of psychometric instruments:

(1) Structural equivalence contributes to the valid use of the instrument in cultural and ethnic contexts other than the one for which the instrument has been developed for. Structural equivalence is a necessary condition for the justification of indirect or direct comparisons between cultural or ethnic groups.
(2) Structural isomorphism is necessary to demonstrate that the same internal structure of the instrument or scales applies to each cultural and/or ethnic group and to the individual levels.

The Bully/Victim Questionnaire (BVQ) by Olweus (1991), was established in one nation many years ago and is widely implemented globally. For many researchers, it provides and assesses the most appropriate definition of bullying and allows actions to be categorized into specific types of bullying and victimization behaviors (e.g., physical, verbal, and relational). There is evidence that it correlates with peer nominations of bullying (Lee and Cornell, 2009) and has good reliability (Breivik and Olweus, 2015). The tool has some limitations where bullies usually do not admit their behavior in self report. Thus, teacher and parental reports may be a valid way to extract this information in addition to the self-report. In addition, although the self-report BVQ is often utilized in cross-national and cross-cultural bullying research, the comparability across different cultural, national or ethnic groups, also referred to as measurement invariance (Widaman and Reise, 1997), has not yet been investigated.

In summary, the literature implies universal, as well as ethnic-specific aspects of bullying behavior, especially when taking diverse types of such behaviors into account. At the moment most of the available evidence cannot be directly compared due to methodological inconsistencies (e.g., utilizing different methods to assess frequency) and divergences in definitions of bullying. These discrepancies led us to conduct a cross-national and cross-ethnic comparative survey amongst five ethnic/national groups in four countries: Germany, Israel (Israeli Jewish and Israeli Palestinians), The Palestinian Authority (the Gaza Strip), and Greece. These ethnic/national groups represent different cultural norms, languages, and different levels of bullying work (e.g., research and anti-bullying intervention) where the same bullying instrument was used. It is an exploratory study with a random sample of convenience. It was felt that selection of the countries in an almost *ad hoc* fashion with this type of research design mimicked the many large and existing cross-cultural studies available today. Very often, countries are chosen to be part of these projects due to a range of random variables such as funding, governmental agendas, available resources and appropriately skilled staff. The aim of the current study is to investigate the extent of comparability of bullying and victimization rates within and between different countries and different ethnic groups including German, Israeli Palestinians, Israeli Jewish, Palestinians in the Gaza Strip, and Greek pupils.

## Materials And Methods

### Design and Sample

The present study is a cross-sectional, cross-national/ethnic comparison between lower secondary school pupils in Germany, Greece, the Gaza Strip in the Palestinian Authority, and Israel (Israeli Jewish and Israeli Palestinians). All samples were stratified according to age. The age range for the whole sample was from 12 to 16 years.

The convenience German sample (see Scheithauer et al., 2006) included two schools consisting of students from two different German federal states: Wittmund, Lower Saxony and the city state of Bremen. The original sample included 2,088 pupils. The sample from Bremen contained a total of 735 students of grades 5–10 from one conventional state secondary school, while the sample from Wittmund, Lower Saxony, represented 1,353 students, attending grades 5–10 of a state secondary school, as it is called “Kooperative Gesamtschule” (cooperative comprehensive school). A final sample of 1,729 German adolescents aged 12–16 years were included in this study.

The Greek sample included a convenient sample from two schools from the greater area of Drama, Greece. From the total sample, 33 parents (10.15%) did not give their written consent, 11 students (3.39%) withdrew and 7 students (2.16%) were not present on the day when the data was collected. Therefore, the final sample consisted of 270 students.

The Palestinian sample from the Gaza Strip included children from four representative areas in the Gaza Strip (Khan-Younis, Mawasy, Beit-Hanon, and Rimal) and from different school levels (primary, junior high school and high school). This is due to the different age groups in each school system. Potential participants were identified in schools and classes in random clusters which represented the Gaza Strip. The study originally included 1,137 students between the ages of 10–18 years. The number of children that completed the bullying questionnaire was 332, from which 266 students between the ages 12–16 years were included in the final sample.

The Israeli sample was administered in one Palestinian and one Jewish lower secondary schools in Israel (see Wolke and Samara, 2004). The Israeli society is composed of a variety of Jewish groups representing approximately 80% of the whole population, while Palestinian Arabs comprise 20%. In general, there are two educational systems in Israel: Jewish (Hebrew as language of instruction) and Arab Palestinians (Arabic as language of instruction), both under the supervision of the Israeli Ministry of Education. A convenient sample from 30 classes in two lower secondary schools in the center district (one from the Arab region and the other from the Jewish region) were chosen to participate in the study. Of these 1,183 pupils, 95 pupils (8%) did not participate as their parents declined permission and a further 167 (14.1%) were not present for data collection. Thus, a final sample of 921 pupils participated.

Table 1 shows the frequency of participants in each ethnic/national group by gender and age. There were no significant differences regarding the distribution of boys and girls in different ages.

**TABLE 1:** Frequency of participants in each ethnic and national group by gender and age.

	**Gender**		**Total**	**Age (mean; SD)**	**Age in years (*N*; %)**				
	
**Ethnic/national group**	**Boys (*N*; %)**	**Girls (*N*; %)**	**(*N*; %)^∗^**		**12**	**13**	**14**	**15**	**16**
Gaza Strip	148 (55.6%)	118 (44.4%)	266 (8.35%)	14.00 (1.57)	70 (26.3%)	41 (15.4%)	51 (19.2%)	28 (10.5%)	76 (28.6%)
Germany	856 (49.5%)	873 (50.5%)	1729 (54.27%)	13.94 (1.39)	374 (21.6%)	316 (18.3%)	369 (21.3%)	383 (22.2%)	287 (16.6%)
Greece	138 (51.1%)	132 (48.9%)	270 (8.47%)	13.80 (1.18)	42 (15.6%)	80 (29.6%)	52 (19.3%)	82 (30.4%)	14 (5.2%)
Israel (Jewish)	217 (48.3%)	232 (51.7%)	449 (14.09%)	13.71 (0.93)	42 (9.4%)	150 (33.4%)	153 (34.1%)	104 (23.2%)	0 (0.0%)
Israel (Palestinians)	231 (48.9%)	241 (51.1%)	472 (14.81%)	13.68 (0.93)	51 (10.8%)	152 (32.2%)	169 (35.8%)	99 (21%)	1 (0.2)
			3186						

*^∗^Out of all samples.*

### Procedure

The procedure was similar for all studies. Prior to the beginning of the research, letters which explained in detail the procedure and the purpose of the study and requested consent for the research were sent to the headteachers of each school. After receiving permission from the headteachers of the schools, letters explaining the aims and the procedure of the studies were sent to the teachers of each class and the children’s parents. Written information about the study and a consent form for parents were passed on via the pupils. The overall aim of this study as well as the questionnaire was explained to the pupils and they were asked to give verbal consent. In addition, the definition of the term “bullying” and patterns of associated aggressive behavior were explained to pupils.

Teams of psychologists and/or social workers in each country carried out the research in each class. All pupils were free to discontinue their participation at any time.

### Ethics Statement

The studies were approved by the ethical committees of the corresponding Universities. The study in Greece was approved by the Ethical Committee of Kingston University London, United Kingdom. The studies in Israel and the Gaza Strip were approved by the Ethical Committee of Hertfordshire University, United Kingdom and the corresponding Ministries of Education in both countries. In Germany, the study was approved by the Institutional Review Board of the University of Bremen. All parents gave written informed consent for their children to participate in the study.

### Instrument

All participants completed the Bully/Victim-Questionnaire (BVQ; Olweus, 1991). The BVQ is an anonymous self-report instrument used to gather information about the extent of bullying. In Germany, an authorized German version (“Fragebogen für Schüler und Schülerinnen ab der 5. Klasse, Form D”) was used. For the Israeli, Greek, and the Gaza Strip samples, the BVQ was translated into Hebrew (for Israeli Jewish), Arabic (for Israeli Palestinians and Palestinians from the Gaza Strip) and Greek (for the sample in Greece) and then back translated to English by qualified translators. Any discrepancies were discussed and rectified for the bullying questions, according to guidelines by van de Vijver and Hambleton (1996).

The questionnaire consists of two parts: things that have been done on purpose to participants and things that participants have done to others on purpose during the last 6 months at school. Each of these two parts contains ten short phrases or questions asking about direct and relational bullying and victimization.

The first five questions were related to victimization: (1) I was hit, kicked, pushed or threatened, (2) I had things taken from me or spoiled; including money, (3) I was made fun of, (4) Children I often play with said that they did not want to play with me (5) Other children told lies or nasty stories about me. The second five questions asked about bullying others: (1) I hit, kicked, pushed or threatened others, (2) I took or spoiled things from others; including money, (3) I made fun of others, (4) I said to children I often play with that I do not want to play with them, (5) I told lies or nasty stories about others.

For all questions, participants were asked how frequently they had experienced or shown these behaviors in the last 6 months. Response options were (0) never (1) only once or twice (2) two or three times a month (3) about once a week or (4) several times a week. The BVQ has been reported to have good validity and reliability (Olweus, 1994).

### Statistical Analysis

Data analysis was conducted with the statistical package software Stata Version 14 and IBM SPSS Statistics 24.

#### Part 1: Differences Between Countries and Ethnic Groups

To assess the relationship of bullying and victimization status according to ethnic/national group two approaches were implemented. We added up the items of bullying to construct a continuous bullying variable and added up the victimization items to construct a continuous victimization variable. Then we performed ANOVA with Bonferroni *post hoc* comparisons between ethnic/national groups.

Secondly, a categorical approach was implemented. For statistical analyses, the first two answer choices for each question were scored as 0 (neutrals) and the others as 1 (frequent bullies or victims). Therefore, children were categorized into four groups: (a) Pure Victims (PV) (those children who have been bullied at least two or three times a month but they have never or only once or twice bullied others in the last 6 months), (b) Pure Bullies (PB) (those children who have bullied others on purpose at least two or three times a month, but they have never or once or twice been victimized in the last 6 months), (c) Bully/Victims (BV) (those children who have been victimized and have bullied others on purpose at least two or three times a month during the last 6 months) and (d) Neutrals (N) (those children who have never, or only once or twice, been victimized or bullied others in the last 6 months). This dichotomous categorization using a cut-off point such as this is based on the core definition of bullying as a repetitive behavior, excluding singular events involving aggressive or violent acts.

Thus, differences in bullying and victimization involvement of each specific item are reported with frequency or cross tables. Bivariate associations between countries were calculated with chi-square-(χ^2^)-statistics (α < 0.05). Additionally, Multinomial Logistic Regression analyses were used to determine the unique effects of ethnic/national group on bullying behavior. The dependent variable (DV) for each logistic regression analysis represents the bullying/victimization subgroups (pure victim, pure bullies, bully/victims) which were compared to neutrals. The odds ratios (OR) and their 95% confidence intervals were determined as an effect measure for data with binary outcomes. The OR displays the relative chance of an outcome’s occurrence (pure victim, pure bullies, bully/victims) in comparison to a reference population (neutral) to investigate differences between each two ethnic/national groups (e.g., German vs. Greek pupils).

#### Part 2: Structural Equivalence and Isomorphism

Evidence of measurement invariance or equivalence was sought using exploratory factor analysis with a matrix of polychoric correlation due to the use of ordinal response variables (Jöreskog, 1994). The analytical approach to test structural equivalence and isomorphism requires several analytical steps, as recommended in Fischer and Fontaine (2010) and Fontaine and Fischer (2010). For these analyses we used the continuous bullying and victimization variables. The testing strategy is presented in two sections.

##### Section 1: Testing for structural equivalence

A hypothesized two-factor structure of the BVQ, “bullying” and “victimization” was tested by computing the individual-level structure (overall factor structure). In this step, any possible national/ethnic differences were ignored, and the validity of factorial structure was tested. In a second step, the applicability of the individual-level structure to each ethnic/national group was tested. Specifically, it was verified whether the hypothesized two-factor structure over all sub-samples (i.e., individual-level structure) is similar to the structure within each ethnic/national group separately using orthogonal Procrustes rotation and evaluating the congruence between factor loadings using Tucker’s coefficient of agreement (Tucker, 1951). To judge similarity, the value of the congruence measure should not be below 0.85 to be indicative of equivalence (Fischer and Fontaine, 2010).

##### Section 2: testing for structural isomorphism

The ethnic/national level association matrix was computed based on the average item scores per ethnic/national group after estimating the size of ethnic/national variation with intra-class correlations (ICCs). Thereby, testing for the hypothesized two-factor structure on the ethnic/national level. Additionally, the ethnic/national-level structure is compared to the individual-level structure by using orthogonal Procrustes rotation and calculations of the congruence measure. Specifically, we tested whether the structure over all samples (i.e., individual-level structure) would apply to the ethnic/national level structure.

## Results

### Part 1: Bullying and Victimization for Each Ethnic/National Group

Tables 2 and 3 show the frequency and the occurrence (according to the answer scale in the last 6 months: never, once or twice, two or three times a month, once a week, several times a week) for each bullying and victimization item for each ethnic/national group. The results show that involvement in different bullying and victimization behaviors varies across ethnic/national groups and occurrences. A general significant difference was found between ethnic/national groups in relation to all bullying and victimization items across the answer scales (*p* < 0.001).

**TABLE 2:** Frequency of each victimization item for each answer scale by ethnic/national groups.

		**Victimization items^∗^**				
		
**Ethnic/national group**	**Answer Scale^+^**	**V (1)**	**V (2)**	**V (3)**	**V (4)**	**V (5)**
Gaza Strip	0	135(50.9%)	159(60%)	189(72.4%)	158(59.6%)	146(55.1%)
	1	78(29.4%)	68(25.7)	41(15.7%)	71(26.8%)	90(34%)
	2	22(8.3%)	22(8.3%)	17(6.5%)	17(6.4%)	15(5.7%)
	2	16(6%)	7(2.6%)	8(3.1%)	9(3.4%)	8(3%)
	4	14(5.3%)	9(3.4%)	6(2.3%)	10(3.8%)	6(2.3%)
Greece	0	139(51.5%)	141(52.2%)	149(55.6%)	157(58.4%)	141(52.6%)
	1	60(22.2%)	62(23%)	66(24.6%)	53(19.7%)	73(27.2%)
	2	52(19.3%)	52(19.3%)	33(12.3%)	44(16.4%)	34(12.7%)
	3	14(5.2%)	12(4.4%)	10(3.7%)	13(4.8%)	17(6.3%)
	4	5(1.9%)	3(1.1%)	10(3.7%)	2(0.7%)	3(1.1%)
Germany	0	1471(89.1%)	1481(90%)	1199(71.8%)	1445(87.4%)	1213(73.1%)
	1	127(7.7%)	136(8.3%)	320(19.2%)	146(8.8%)	332(20%)
	2	22(1.3%)	14(0.9%)	63(3.8%)	20(1.2%)	50(3%)
	3	11(0.7%)	7(0.4%)	26(1.6%)	19(1.1%)	34(2%)
	4	20(1.2%)	7(0.4%)	63(3.8%)	23(1.4%)	31(1.9%)
Israel (Jewish)	0	350(78%)	350(78%)	322(71.7%)	319(71%)	273(60.9%)
	1	76(16.9%)	77(17.1%)	77(17.1%)	80(17.8%)	120(26.8%)
	2	15(3.3%)	11(2.4%)	27(6%)	22(4.9%)	29(6.5%)
	3	7(1.6%)	8(1.8%)	13(2.9%)	12(2.7%)	12(2.7%)
	4	1(0.2%)	3(0.7)	10(2.2%)	16(3.6%)	14(3.1%)
Israel (Palestinians)	0	334(70.8%)	360(76.3%)	362(76.7%)	375(79.4%)	297(62.9%)
	1	91(19.3%)	76(16.1%)	52(11%)	62(13.1%)	120(25.4%)
	2	23(4.9%)	14(3%)	13(2.8%)	15(3.2%)	25(5.3%)
	3	13(2.8%)	13(2.8%)	34(7.2%)	12(2.5%)	9(1.9%)
	4	11(2.3%)	9(1.9%)	11(2.3%)	8(1.7%)	21(4.4%)

*^∗^Victimization items with overall significant comparisons between ethnic/national groups: (1) I was hit, kicked, pushed or threatened [χ^2^ = 464.12 (16, 3107), *p* < 0.001], (2) I had things taken from me or spoiled; including money [χ^2^ = 442.22 (16, 3101), *p* < 0.001], (3) I was made fun of [χ^2^ = 120.34 (16, 3121), *p* < 0.001], (4) Children I often play with said that they did not want to play with me [χ^2^ = 305.39 (16, 3108), *p* < 0.001], (5) Other children told lies or nasty stories about me [χ^2^ = 130.45 (16, 3113), *p* < 0.001]. ^+^Answer scale: (0) never (1) only once or twice (2) two or three times a month (3) about once a week or (4) several times a week.*

**TABLE 3:** Frequency of each bullying item for each answer scale by ethnic/national groups.

		**Bullying items^∗^**				
		
**Ethnic/national group**	**Answer Scale^+^**	**B (1)**	**B (2)**	**B (3)**	**B (4)**	**B (5)**
Gaza Strip	0	190(72.2%)	228(86.4%)	207(79.9%)	189(71.3%)	237(78.4%)
	1	48(18.3%)	19(7.2%)	33(12.7%)	51(19.2%)	20(7.6%)
	2	10(3.8%)	9(3.4%)	13(5%)	14(5.3%)	4(1.5%)
	3	6(2.3%)	4(1.5%)	4(1.5%)	6(2.3%)	0(0%)
	4	9(3.4%)	4(1.5%)	2(0.8%)	5(1.9%)	2(0.8%)
Greece	0	144(53.3%)	171(63.6%)	172(63.9%)	169(62.6%)	168(62.5%)
	1	60(22.2%)	47(17.5%)	60(22.3%)	54(20%)	59(21.9%)
	2	57(21.1%	37(13.8%)	27(10%)	33(12.2%)	35(13%)
	3	5(1.9%)	12(4.5%)	7(2.6%)	13(4.8%)	5(1.9%)
	4	4(1.5%)	2(0.7%)	3(1.1%)	1(0.4%)	2(0.7%)
Germany	0	1418(86%)	1572(95.7%)	931(55.4%)	1212(73.1%)	1435(87.5%)
	1	153(9.3%)	32(1.9%)	485(28.8%)	292(17.6%)	137(8.4%)
	2	16(1%)	8(0.5%)	96(5.7%)	50(3%)	19(1.2%)
	3	16(1%)	6(0.4%)	74(4.4%)	37(2.2%)	20(1.2%)
	4	45(2.7%)	24(1.5%)	96(5.7%)	76(4%)	29(1.8%)
Israel (Jewish)	0	356(79.3%)	405(90.2%)	328(73.2%)	317(70.6%)	352(78.4%)
	1	66(14.7%)	26(5.8%)	68(15.2%)	94(20.9%)	65(14.5%)
	2	15(3.3%)	11(2.4%)	31(6.9%)	26(5.8%)	21(4.7%)
	3	8(1.8%)	4(0.9%)	9(2%)	6(1.3%)	4(0.9%)
	4	4(0.9%)	3(0.7%)	12(2.7%)	6(1.3%)	7(1.6%)
Israel (Palestinians)	0	361(76.6%)	433(91.7%)	376(80%)	379(80.3%)	406(86%)
	1	67(14.2%)	18(3.8%)	51(10.9%)	58(12.3%)	41(8.7%)
	2	22(4.7%)	13(2.8%)	17(3.6%)	20(4.2%)	16(3.4%)
	3	7(1.5%)	3(0.6%)	15(3.2%)	7(1.5%)	4(0.8%)
	4	14(3%)	5(1.1%)	11(2.3%)	8(1.7%)	5(1.1%)

*^∗^Bullying items with overall significant comparisons between ethnic/national groups: (1) I hit, kicked, pushed or threatened others [χ^2^ = 333.84 (16, 3101), *p* < 0.001], (2) I took or spoiled things from others; including money [χ^2^ = 363.39 (16, 3096), *p* < 0.001], (3) I made fun of others [χ^2^ = 188.60 (16, 3128), *p* < 0.001], (4) I said to children I often play with that I do not want to play with them [χ^2^ = 96.68 (16, 3114), *p* < 0.001], (5) I told lies or nasty stories about others [χ^2^ = 192.37 (16, 3093), *p* < 0.001]. ^+^Answer scale: (0) never (1) only once or twice (2) two or three times a month (3) about once a week or (4) several times a week.*

Looking at the sum of the victimization items and bullying items, results from ANOVA with Bonferroni *post hoc* revealed that there are significant differences between ethnic/national groups. Greek pupils were more likely to be involved in bullying behaviors compared to all other ethnic groups (*p* < 0.001). On the other hand, Greece and Gaza adolescents were significantly more likely to be involved in victimization compared to all other ethnic/national groups (*p* < 0.001) and Israeli Jewish and Israeli Palestinians were significantly more likely to be involved in victimization compared to German adolescents (*p* < 0.001) (see Table 4).

**TABLE 4:** Overall bullying subgroups within each ethnic/national group^∗^ and mean and standard deviation for the sum of the bullying and victimization items for each ethnic/national group^†^.

	**Israel (Jewish) (*N*: 449)**	**Israel (Palestinians) (*N*: 472)**	**Gaza Strip (*N*: 266)**	**Greece (*N*: 270)**	**Germany (*N*: 1,729)**
Neutrals	265(59%)	294(62.3%)	153(57.5%)	105(38.9%)	1177(68.1%)
Pure victims	90(20%)	85(18%)	61(22.9%)	59(21.9%)	173(10%)
Pure bullies	69(15.4%)	44(9.3%)	14(5.3%)	39(14.4%)	264(15.3%)
Bully/victims	25(5.6%)	49(10.4%)	38(14.3%)	67(24.8%)	115(6.7%)
Sum of bullying items (mean, SD)	1.67(2.43)	1.46(2.59)	1.58(2.47)	3.08(3.43)	1.76(3.02)
Sum of victimization items (mean, SD)	2.16(2.66)	2.25(3.05)	3.23(3.37)	3.83(3.71)	1.34(2.44)

*^∗^Specific comparisons between ethnic/national groups using the overall bullying variable including the four subgroups: neutrals, pure victims, pure bullies, and bully/victims: Israel (Jewish) vs. Israel (Palestinians): [χ^2^ = 14.40 (3, 921), *p* < 0.01]; Israel (Jewish) vs. Gaza Strip: [χ^2^ = 29.82 (3, 715), *p* < 0.001]; Israel (Jewish) vs. Greece: [χ^2^ = 62.45 (3, 719), *p* < 0.001]; Israel (Jewish) vs. Germany: [χ^2^ = 34.81 (3, 2178), *p* < 0.001]; Israel (Palestinians) vs. Gaza Strip: [χ^2^ = 8.49 (3, 738), *p* < 0.05]; Israel (Palestinians) vs. Greece: [χ^2^ = 45.71 (3, 742), *p* < 0.001]; Israel (Palestinians) vs. Germany: [χ^2^ = 38.41 (3, 2201), *p* < 0.001]; Gaza Strip vs. Greece: [χ^2^ = 28.74 (3, 536), *p* < 0.001]; Gaza Strip vs. Germany: [χ^2^ = 70.78 (3, 1995), *p* < 0.001]; Greece vs. Germany: [χ^2^ = 143.98 (3, 1999), *p* < 0.001]. ^†^Overall difference between ethnic/national groups in relation to sum of bullying items: *F* = 15.73 (4, 3150), *p* < 0.001, η^2^ = 0.020; overall difference between ethnic/national groups in relation to sum of victimization items: *F* = 67.39 (4, 3150), *p* < 0.001, η^2^ = 0.079.*

**FIGURE 1:**
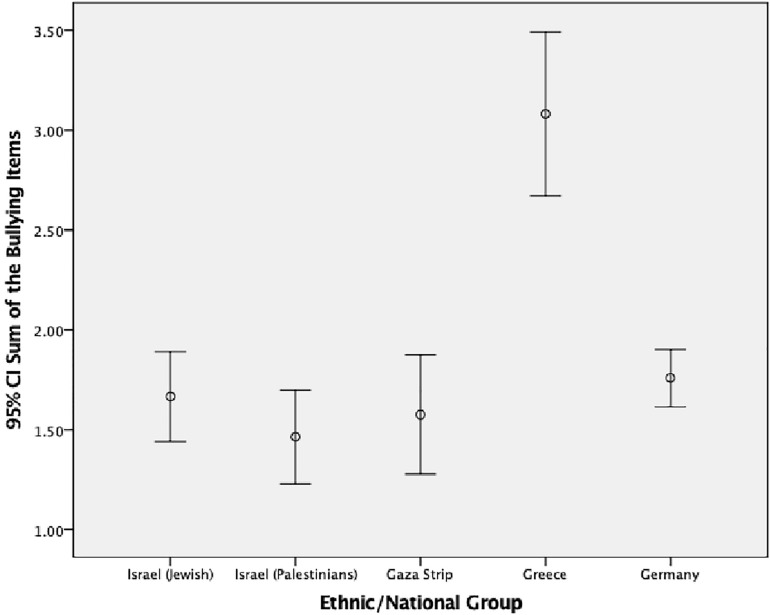
Mean and 95% confidence interval for involvement in bullying others by ethnic/national group.

**FIGURE 2:**
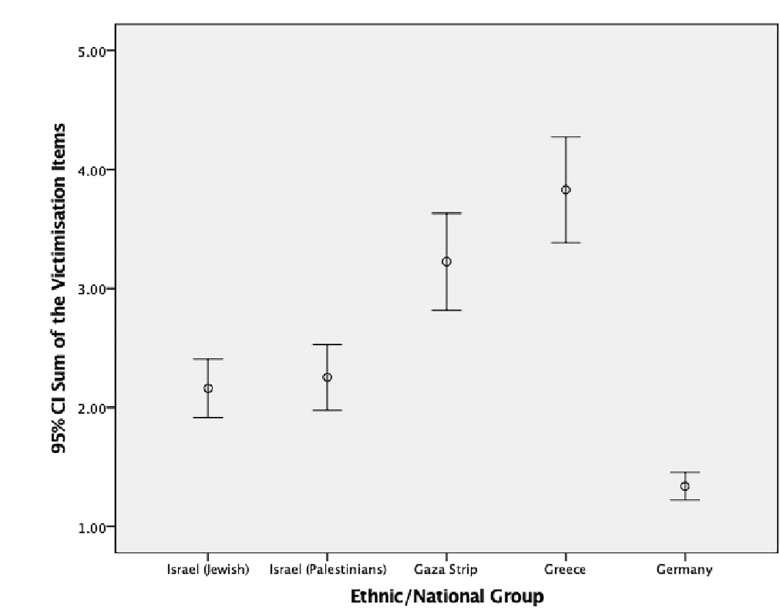
Mean and 95% confidence interval for involvement in victimization by ethnic/national group.

We also looked at differences between ethnic/national groups using the overall bullying variable including the four subgroups: neutrals, pure victims, pure bullies, and bully/victims. Table 4 shows the prevalence of each subgroup for each ethnic/national group separately. When looking at bullying subgroups for each ethnic/national group, crosstabs analysis showed overall significant differences between each ethnic/national group with the other ethnic/national groups (10 comparisons in total) (Israeli Jewish vs. Israeli Palestinians: *p* < 0.01; Israeli Palestinians vs. Palestinians from the Gaza Strip: *p* < 0.05; the remaining comparisons: *p* < 0.001) (see Table 4 and Figures 1, 2).

Multinomial logistic regressions were performed to see the specific differences between each two ethnic/national groups in relation to each bullying subgroup where the reference point of comparison was the neutral subgroup (Table 5 also shows the frequency of each bullying subgroup in comparison to the neutral group for each ethnic/national group). The results of the multinomial logistic regressions comparisons were as follows:

**TABLE 5:** The frequency of each bullying subgroup in comparison to the neutral group.

	**Israel (Jewish)**	**Israel (Palestinians)**	**Gaza Strip**	**Greece**	**Germany**
Neutrals (comparison group)	*n*: 265	*n*: 294	*n*: 153	*n*: 105	*n*: 1,177
Pure victims	90/355 (25.35%)	85/379 (22.43%)	61/214 (28.50%)	59/164 (35.98%)	173/1350 (12.81%)
Pure bullies	69/334 (20.66%)	44/338 (13.02%)	14/167 (8.38%)	39/144 (27.08%)	264/1441 (18.32%)
Bully/victims	25/290 (8.62%)	49/343 (14.29%)	38/191 (19.90%)	67/172 (38.95%)	115/1292 (8.90%)

#### Israeli Jewish vs. Israeli Palestinians

The overall model was significant [χ^2^ = 14.58 (3, 921), *p* < 0.01]. Israeli Jewish were more likely to be involved in pure bullying others in comparison to Israeli Palestinians (OR: 1.74, 95% CI: 1.15–2.63, *p* < 0.01), while Israeli Palestinians were more likely to be involved as bully/victims in comparison to Israeli Jewish (OR: 1.77, 95% CI: 1.06–2.94, *p* < 0.05).

#### Israeli Jewish vs. Palestinians in the Gaza Strip

The overall model was significant [χ^2^ = 31.06 (3, 715), *p* < 0.001]. Israeli Jewish were more likely to be involved in pure bullying others in comparison to Palestinians from the Gaza Strip (OR: 2.85, 95% CI: 1.55–5.23, *p* < 0.01), while Palestinians from the Gaza Strip were more likely to be involved as bully/victims in comparison to Israeli Jewish (OR: 2.63, 95% CI: 1.53–4.52, *p* < 0.001).

#### Israeli Jewish vs. Greek Children

The overall model was significant [χ^2^ = 61.33 (3, 719), *p* < 0.001]. Greek children were more likely to be pure victims (OR: 1.65, 95% CI: 1.11–2.46, *p* < 0.05) and bully/victims (OR: 6.76, 95% CI: 4.05–11.24, *p* < 0.001) compared to Israeli Jewish children.

#### Israeli Jewish vs. German Children

The overall model was significant [χ^2^ = 31.39 (3, 2178), *p* < 0.001]. Israeli Jewish children were more likely to be pure victims (OR: 2.31, 95% CI: 1.73–3.08, *p* < 0.001) compared to German children.

#### Israeli Palestinians vs. Palestinians From the Gaza Strip Children

The overall model was significant [χ^2^ = 8.63 (3, 738), *p* < 0.05] but no specific differences between the two groups in relation to the bullying subgroups were found.

#### Israeli Palestinians vs. Greek Children

The overall model was significant [χ^2^ = 45.35 (3, 742), *p* < 0.001]. Greek children were more likely to be pure victims (OR: 1.94, 95% CI: 1.30–2.90, *p* < 0.01), bullies (OR: 2.48, 95% CI: 1.53–4.03, *p* < 0.001) and bully/victims (OR: 3.83, 95% CI: 2.49–5.88, *p* < 0.001) in comparison to Israeli Palestinian children.

#### Israeli Palestinians vs. German Children

The overall model was significant [χ^2^ = 36.80 (3, 2201), *p* < 0.001]. Israeli Palestinians were more likely to be pure victims (OR: 1.97, 95% CI: 1.47–2.63, *p* < 0.001) and bully/victims (OR: 1.71, 95% CI: 1.19–2.44, *p* < 0.01) in comparison to German children, while German children were more likely to be involved as bullies (OR: 1.50, 95% CI: 1.06–2.11, *p* < 0.05).

#### Palestinians From the Gaza Strip vs. Greek Children

The overall model was significant [χ^2^ = 29.38 (3, 536), *p* < 0.001]. Greek children were more likely to be involved in bullying as bullies (OR: 4.06, 95% CI: 2.10–7.87, *p* < 0.001) and bully/victims (OR: 2.57, 95% CI: 1.61–4.12, *p* < 0.001) in comparison to Palestinian children from the Gaza Strip.

#### Palestinians From the Gaza Strip vs. German Children

The overall model was significant [χ^2^ = 66.35 (3, 1995), *p* < 0.001]. Palestinian children from the Gaza Strip were more likely to be pure victims (OR: 2.71, 95% CI: 1.94–3.80, *p* < 0.001) and bully/victims (OR: 2.54, 95% CI: 1.70–3.81, *p* < 0.001) in comparison to German children, while German children were more likely to be involved as bullies (OR: 2.45, 95% CI: 1.39–4.31, *p* < 0.01) in comparison to Palestinian children from the Gaza Strip.

#### Greek vs. German Children

The overall model was significant [χ^2^ = 120.96 (3, 1999), *p* < 0.001]. Greek children were more likely to be pure victims (OR: 3.82, 95% CI: 2.68–5.46, *p* < 0.001), bullies (OR: 1.66, 95% CI: 1.12–2.45, *p* < 0.05) and bully/victims (OR: 6.53, 95% CI: 4.55–9.37, *p* < 0.001) in comparison to German children.

### Part 2: Structural Equivalence and Isomorphism

**FIGURE 3:**
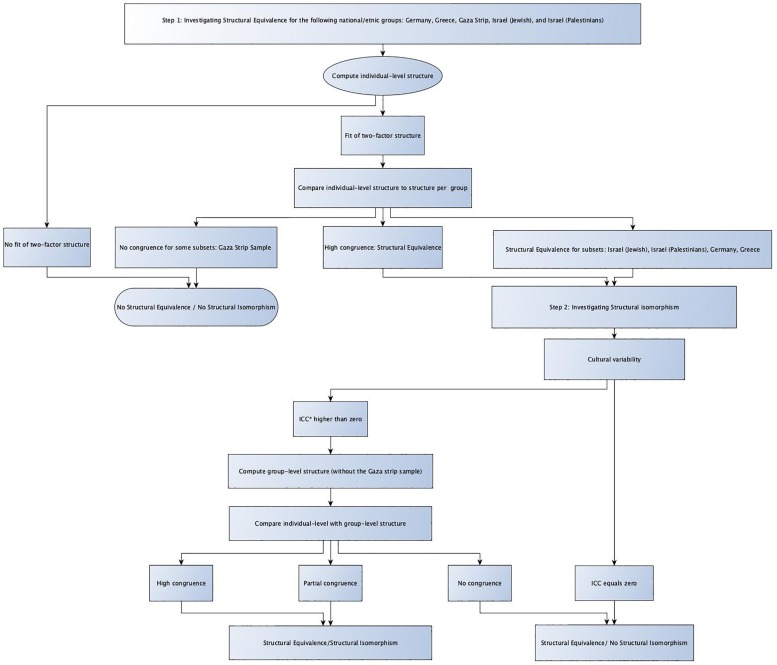
Overview of steps for the analysis of structural equivalence and structural isomorphism (Fischer and Fontaine, 2010; Fontaine and Fischer, 2010). ^∗^ICC = Intra-class correlation.

The above results revealed significant differences between ethnic/national groups in relation to involvement in bullying behaviors as bullies, victims or bully/victims. In this section, we will perform extra analysis to confirm whether the above results are valid and whether the comparisons between ethnic/national groups in relation to bullying and victimization is adequate and represent reality. In addition, we will test whether the use of these specific items represent two distinct behaviors (bullying and victimization) in each ethnic/national group. Thus, we performed structural equivalence and isomorphism analyses. As those two concepts are hierarchically ordered – the investigation of structural equivalence gives necessary but insufficient information and functions as analytical basis for isomorphism. Results for each section are explained in detail below and Figure 3 for overview of the analytical steps.

#### Section 1: Testing for Structural Equivalence

At the individual level, the expected two-factorial structure of the BVQ, “bullying” and “victimization” clearly emerged (see Table 6). Subsequently, the factor structure of each cultural/national sample was orthogonally Procrustes rotated toward the individual level structure and the congruence measure calculated for each factor per ethnic/national group. For most ethnic/national groups, the Tucker’s coefficient of agreement exceeded 0.85 or even 0.95, with the exception of the sample from the Gaza Strip, which showed congruence value of 0.74 (victimization) and 0.65 (bullying). This finding supports the structural equivalence with the exception of the Gaza Strip sample.

**TABLE 6:** The individual level structure with factor loading for the hypothesized two-factor structure of the BVQ (*N* = 3,186).

		**Bullying**	**Victimization**
	**Victimization items**		
1	I was hit, kicked, pushed or threatened	0.1175	**0.7308**
2	I had things taken from me or spoiled; including money	0.1486	**0.6256**
3	I was made fun of	0.0213	**0.6464**
4	Children I often play with said that they did not want to play with me	−0.0747	**0.7484**
5	Other children told lies or nasty stories about me	−0.0433	**0.7090**
	**Bullying items**		
1	I hit, kicked, pushed or threatened others	**0.7450**	0.0928
2	I took or spoiled things from others; including money	**0.7816**	0.1182
3	I made fun of others	**0.7231**	−0.1458
4	I said to children I often play with that I do not want to play with them	**0.6857**	−0.0959
5	I told lies or nasty stories about others	**0.6920**	0.0979

*Bold: Factor loadings above 0.40.*

#### Section 2: Testing for Structural Isomorphism

The individual items of the BVQ varied sufficiently between cultural/national groups. The intra-class correlation coefficient ranged from 0.016 to 0.11. The Gaza Strip sample was excluded from further analysis, due to the lack of structural equivalence. Subsequently, exploratory factor analysis on the ethnic/national level structure revealed a one-factor structure with congruence measure below 0.85. Thus, no evidence was found for structural isomorphism. Therefore, no further direct comparisons of the cultural/national samples are justified.

## Discussion

Our study set out to examine the validity of cross-ethnic and cross-national comparisons in relation to bullying and victimization rates using the same instrument (i.e., the BVQ). First, we compared the different ethnic/national groups and the results revealed significant differences in relation to involvement in bullying and victimization behaviors. The results showed that Greek children were more likely to be involved in bullying as pure victims in comparison to Israeli Jewish, Israeli Palestinian and German children, and as bullies and bully/victims in comparison to Israeli Palestinians, Palestinians in the Gaza Strip, Israeli Jewish and German children. The Israeli Jewish sample, on the other hand, were more likely to be involved in bullying as pure bullies in comparison to Israeli Palestinians and Palestinians in the Gaza Strip, and as victims in comparison to German children. Both Israeli Palestinians and Palestinians in the Gaza Strip were more likely to be involved in bullying as victims and bully/victims in comparison to German children, while German children were more likely to be involved as bullies. Finally, Israeli Palestinians and Palestinians in the Gaza Strip were more likely to be involved as bully/victims in comparison to Israeli Jewish. No differences were found between Israeli Palestinians and Palestinians from the Gaza Strip in relation to the bullying subgroups. The odds ratios ranged from 1.65 to 6.53, which indicated that differences are not equal between ethnic groups.

Nonetheless, do the above results mean that each specific difference found represent reality? Or to put it another way, can we say that the specific ethnic groups are more or less likely to be a bully, victim or bully/victim in comparison to the other ethnic group using one standard questionnaire? In order to answer these questions, we deemed it necessary to perform structural equivalence and isomorphism analyses to examine the use of the bullying questionnaire within each ethnic group and to assess whether comparability is valid across the same groups. We initially verified whether the hypothesized two-factor structure of the BVQ, “bullying” and “victimization” over all sub-samples (i.e., individual-level structure) was similar to the structure within each ethnic group separately. We then tested whether the structure over all samples (i.e., individual-level structure) would apply to the ethnic level structure. This was necessary to investigate the usefulness of our instrument and indeed, to determine if the initial conclusions drawn regarding the prevalence of bullying and victimization were appropriate.

The results found that at the individual level, the expected two-factorial structure of the BVQ, “bullying” and “victimization” clearly emerged. This finding supports the internal structure equivalence for each ethnic/national group with the exception of the Gaza Strip sample. Secondly, the exploratory factor analysis on the ethnic level structure revealed a one-factor structure with congruence measure below 0.85. Thus, no evidence was found for structural isomorphism and no further direct comparisons of the ethnic/national samples are justified. Thus, the structural equivalence and isomorphism analyses disapprove and invalidate the first section of results where we report significant differences between different ethnic/national groups (even within the same country, i.e., Israel). Also, the results show that the bullying questionnaire did not generate distinct bullying and victimization factors for the Gaza Strip sample.

Bullying is a recognized form of problematic behavior that is investigated worldwide in most cultures, ethnic groups and countries with shared and similar characteristics, different types and forms, and nature (Smith et al., 2016a). Research on cross-national and cross-ethnic comparisons on bullying to date relied on specific methodological approaches. Comparisons on rates and prevalence of specific bullying items or forms are often established using standard questionnaires that have been translated into appropriate languages. Although these studies can give some indication of differences between cultures or ethnic groups, the results reported here confirm that we need to treat these findings with caution. Statistical data analysis is also considered as a tool to determine whether cross-national or cross-ethnic comparison is valid and represent true differences and variations between cultures or even between ethnic groups within the same country.

Of note, the first statistical methodology, testing for structural equivalence, where we found that the bullying tool used in the five studies has two distinct behaviors of victimization and bullying (except for the Gaza sample), indicates that the bullying questionnaire can be used to measure bullying and/or victimization within each ethnic/national group separately. For the Gaza Strip sample, the testing revealed that there are no distinct groups of bullying versus victimization that can be extracted from the items used. This can be interpreted by different reasons. Firstly, the political situation and the war in the Gaza Strip, where the whole population has been exposed to traumatic events (e.g., house demolition, killing of a relative, injuries) and to a siege since 2007, may thus make bullying questions and items seem like small events in comparison to these war events (Altawil et al., 2008; Abdeen et al., 2018; El-Khodary and Samara, 2018, 2019). Secondly, there is a need for further analysis for this specific sample, where we should look at different types and forms of bullying (physical, verbal, relational) rather than general bullying and victimization. Thirdly, this could also be related to the difficult economic situation in the Gaza Strip compared to the other four samples.

In contrast, when applying the structural isomorphism testing, direct comparisons of the ethnic/national samples are not justified. The results raise awareness of how easily comparisons across groups can lead to spurious results. There is thus a need for preliminary analysis for each construct before evaluating group differences. Even within the same country (i.e., Israeli Palestinians and Jewish) comparisons cannot be conducted due to lack of evidence for structural isomorphism. Children and adolescents may perceive the meaning of the bullying items differently and thus comparisons may not reflect true differences or similarities. Furthermore, translating a specific questionnaire to other languages necessitates different validity tests that need to be performed to make sure that the questionnaire is measuring what it is intended to measure. This could also be due to procedural issues such as how the studies were performed in different countries and amongst different ethnic/national groups, how much the researchers were involved, and the level of explanation that the participants received about the bullying items. Finally, country differences such as socioeconomic inequality (Chaux et al., 2009) or cultural values (e.g., individualism–collectivism; Smith and Robinson, 2019) may differ from one study to another.

Several limitations and issues warranting further research need to be considered when reviewing these results. First, these were convenience samples of different sizes and may not be nationally representative in some samples. A larger sample might provide more illuminating results (e.g., the Gaza Strip). Another limitation of this study is that it relies on self−reports and not on behavioral measures of bullying. As such, the risk of selection effects and biases have to be taken into account. Current limitations of the methods must also be acknowledged. For example, the conventional classification approach for bullying resulting in the common classes of “pure victim,” “pure bully,” “bully-victim,” and “neutrals” might overestimate involvement (see Schultze-Krumbholz et al., 2015 for further information). As evident in the current manuscript, there are a range of methodological shortcomings with this approach (translation and perception of the word bullying, different designs, reference time frame, answer scales, cut-off points or data analysis approaches; Sabella et al., 2013; Smith, 2014; Foody et al., 2017). More advanced methods to investigate measurement invariance like Multigroup Confirmatory Factor Analysis (MGCFA, e.g. Jovanović et al., 2019) or Multigroup Latent Class Analysis (MGLCA, e.g. Eid et al., 2003) are advisable and should be prioritized in future research. Nevertheless, we found the exploratory factor analysis, as recommended by Fischer and Fontaine (2010), more suitable in respect to the instrument used (i.e., BVQ) despite the restricted sample size on an individual and cultural level.

## Conclusion

The statistical methodologies used in this study showed the importance of the methodological approach that is adapted when comparing bullying and victimization across different cultures and ethnic groups. We need to consider different issues when comparing different countries, cultures, and ethnic groups (between and within countries). Furthermore, cultural differences in interrupting and perceiving peer bullying and/or victimization situations, and the internal and the external validity of any study need to be taken into account to be able to compare between different ethnic/national groups. Countries differ on many characteristics like educational policies, personal beliefs, attitudes, values, and so on. Other factors that need to be taken into account are linguistic issues related to the translation and definition of bullying in different cultures, and measurements invariance that could be related to age and gender differences. Future analysis should also look at the different forms of bullying and victimization, including physical, verbal, relational, and cyber bullying. In addition, a failure to demonstrate invariance can be helpful to make conclusions about how different groups interpret the same construct. Some constructs are simply experienced so differently across various groups.

The results of the current study raised a fundamental demand that different aspects need to be taken into account when comparing bullying and victimization between and within countries. This study is a contribution to the discussion of whether and how study results from different nations and/or cultures can be compared. Although standards have been defined for cross-cultural research for some time (e.g., Matsumoto and Van de Vijver, 2010), these standards have not yet been become part of cross-national bullying research.

Bullying is a concern for children, parents, schools, and practitioners (Samara et al., 2017). These groups, as well as policy makers, educational practitioners, and researchers should take into account the current results when attempting to compare between different ethnic/national groups or even across schools. The current results also call into question the common practice of adopting any given anti-bullying intervention or prevention program from another cultural context to another. The results presented here suggest that their utility may also depend on potential cultural or ethnic values and perceptions (Samara and Smith, 2008; Smith et al., 2008, 2012).

## Data Availability

The datasets for this manuscript are not publicly available because they are used in other ongoing studies for publication. Requests to access the datasets should be directed to the authors of the manuscript (MS: M.Samara@kingston.ac.uk and HS: herbert.scheithauer@fu-berlin.de).

## Ethics Statement

This study was carried out in accordance with the recommendations of British Psychological Society Guidelines with written informed consent from all subjects. All subjects gave written informed consent in accordance with the Declaration of Helsinki. The study in Greece was approved by the Ethical Committee of the University of Kingston London, United Kingdom. The studies in Israel and the Gaza Strip were approved by the Ethical Committee of Hertfordshire University, United Kingdom and the corresponding Ministries of Education in both countries. In Germany, the survey was conducted in accordance with the guidelines of the Institutional Review Board of the University of Bremen.

## Author Contributions

MS, HS, and KG contributed to the conception and design of the study. KG and MS organized the database and performed the statistical analysis. All authors contributed to the acquisition and interpretation of data for the work and all authors drafted the work and revised it critically for important intellectual content and approved the submitted version.

## Conflict of Interest Statement

The authors declare that the research was conducted in the absence of any commercial or financial relationships that could be construed as a potential conflict of interest.
